# Distinct aging profiles of CD8^+^ T cells in blood versus gastrointestinal mucosal compartments

**DOI:** 10.1371/journal.pone.0182498

**Published:** 2017-08-23

**Authors:** Jeffrey Dock, Christina M. Ramirez, Lance Hultin, Mary Ann Hausner, Patricia Hultin, Julie Elliott, Otto O. Yang, Peter A. Anton, Beth D. Jamieson, Rita B. Effros

**Affiliations:** 1 Department of Pathology and Laboratory Medicine, David Geffen School of Medicine, University of California-Los Angeles, Los Angeles, CA, United States of America; 2 Department of Biostatistics, Fielding School of Public Health, University of California-Los Angeles, Los Angeles, CA, United States of America; 3 Division of Hematology and Oncology, Department of Medicine, David Geffen School of Medicine, University of California-Los Angeles, Los Angeles, CA, United States of America; 4 UCLA AIDS Institute, David Geffen School of Medicine at UCLA, United States of America; 5 Department of Epidemiology, Fielding School of Public Health, University of California-Los Angeles, Los Angeles, CA, United States of America; 6 Division of Digestive Diseases, Department of Medicine, David Geffen School of Medicine, University of California-Los Angeles, Los Angeles, CA, United States of America; 7 Division of Infectious Diseases, Department of Medicine, David Geffen School of Medicine, University of California-Los Angeles, Los Angeles, CA, United States of America; 8 Department of Microbiology Immunology & Molecular Genetics, David Geffen School of Medicine, University of California-Los Angeles, Los Angeles, CA, United States of America; 9 AIDS Healthcare Foundation, Los Angeles, CA, United States of America; Emory University School of Medicine, UNITED STATES

## Abstract

A hallmark of human immunosenescence is the accumulation of late-differentiated memory CD8^+^ T cells with features of replicative senescence, such as inability to proliferate, absence of CD28 expression, shortened telomeres, loss of telomerase activity, enhanced activation, and increased secretion of inflammatory cytokines. Importantly, oligoclonal expansions of these cells are associated with increased morbidity and mortality risk in elderly humans. Currently, most information on the adaptive immune system is derived from studies using peripheral blood, which contains approximately only 2% of total body lymphocytes. However, most lymphocytes reside in tissues. It is not clear how representative blood changes are of the total immune status. This is especially relevant with regard to the human gastrointestinal tract (GALT), a major reservoir of total body lymphocytes (approximately 60%) and an anatomical region of high antigenic exposure. To assess how peripheral blood T cells relate to those in other locations, we compare CD8^+^ T cells from peripheral blood and the GALT, specifically rectosigmoid colon, in young/middle age, healthy donors, focusing on phenotypic and functional alterations previously linked to senescence in peripheral blood. Overall, our results indicate that gut CD8^+^ T cells show profiles suggestive of greater differentiation and activation than those in peripheral blood. Specifically, compared to blood from the same individual, the gut contains significantly greater proportions of CD8^+^ T cells that are CD45RA^-^ (memory), CD28^-^, CD45RA^-^CD28^+^ (early memory), CD45RA^-^CD28^-^ (late memory), CD25^-^, HLA-DR^+^CD38^+^ (activated) and Ki-67^+^ (proliferating); *ex vivo* CD3^+^ telomerase activity levels are greater in the gut as well. However, gut CD8^+^ T cells may not necessarily be more senescent, since they expressed significantly lower levels of CD57 and PD-1 on CD45RO^+^ memory cells, and had *in vitro* proliferative dynamics similar to that of blood cells. Compartment-specific age-effects in this cohort were evident as well. Blood cells showed a significant increase with age in proportion of HLA-DR^+^38^+^, Ki-67^+^ and CD25^+^ CD8^+^ T cells; and an increase in total CD3^+^ *ex-vivo* telomerase activity that approached significance. By contrast, the only age-effect seen in the gut was a significant increase in CD45RA^-^ (memory) and concurrent decrease in CD45RA^+^CD28^+^ (naïve) CD8^+^ T cells. Overall, these results indicate dynamics of peripheral blood immune senescence may not hold true in the gut mucosa, underscoring the importance for further study of this immunologically important tissue in evaluating the human immune system, especially in the context of chronic disease and aging.

## Introduction

Immunosenescence, the age-associated decline in immune competence, is characterized by a wide range of functional and phenotypic alterations to the immune system [1, 2]. This constellation of features is associated with increased susceptibility to infectious diseases and cancer, reduced effectiveness of vaccination, increased autoimmune phenomena, tissue damage due to dysregulated inflammation, and ultimately, higher mortality risk [3–6]. One hallmark of immunosenescence is the accumulation of late-differentiated memory CD8^+^ T cells with features of replicative senescence, such as inability to proliferate, absence of CD28 gene and protein expression, shortened telomeres, enhanced activation and increased secretion of inflammatory cytokines [7, 8]. The abundance of oligoclonal expansions of these late differentiated memory CD8^+^ T cells is associated with restriction in the overall CD8^+^ T cell repertoire [9, 10], and is correlated with morbidity and mortality in the elderly [9, 11, 12].

An important caveat regarding research on human immunosenescence is that most studies have been performed on peripheral blood, which contains only 2% of total body lymphocytes. By contrast, gut-associated lymphoid tissue (GALT) contains 40–65% of lymphocytes and is an area of high antigenic exposure, but has rarely been investigated [13, 14]. Moreover, there is minimal information on the relationship of CD8^+^ T cells within the GALT and peripheral blood, and how the composite of these two populations contributes to immunosenescence.

In the few studies that have compared GALT and peripheral blood cells, several phenotypic differences have been documented in the T cell compartment. Most notably, studies comparing peripheral blood and intestinal lamina propria T cells indicated that, whereas most peripheral blood T cells were naïve (CD45RO^-^) and non-activated, mucosal T cells are generally in a more activated state and are mainly (>98%) memory (CD45RO^+^) [15]. Also, there is evidence indicating gut CD4^+^ T cells are more activated than their peripheral blood counterparts, and show greater susceptibility to HIV infection *in vitro* [16]. Other research on the mucosal immune system indicates that, compared to CD8^+^ T cells in peripheral blood, those present in breast milk are more abundant and show increased expression of activation and senescence-related markers [17]. These observations of phenotypic and possibly functional differences across anatomical regions underscore the need for a more comprehensive analysis of the senescence profile of gut CD8^+^ T cells.

In the current study, we compare CD8^+^ T cells from peripheral blood and GALT (taken from rectosigmoid colon biopsies) of 39 healthy, young/middle age donors (range: 24–67 years old; mean age: 40.8; male (n = 35), female (n = 3), MTF (n = 1)), focusing on phenotypic and functional alterations that had been previously linked to senescence in peripheral blood. These initial studies intentionally avoided older individuals, many of whom have chronic diseases that could confound the analysis. We examined cell surface markers that are associated with age-related senescence (and in many cases increased morbidity and mortality risk), including CD45RA, CD28, CD57, PD-1 and CD25. We also analyzed markers of activation and homeostatic proliferation, including HLA-DR, CD38, intracellular Ki-67 and *ex vivo* telomerase activity, as chronic cellular activation *in vivo* is believed to be a precursor to ongoing differentiation and, ultimately, replicative senescence [2]. In addition, we evaluated the *in vitro* proliferative capacity of blood and gut CD8 T cells stimulated via the TCR; as poor CD8^+^ T cell proliferative capacity is a hallmark of immune senescence and is also correlated with increased morbidity and mortality risk [18, 19]. Finally, within our relatively young sample population, we examined whether there was any differential age-effect on senescence parameters in blood versus gut T cells. Previous studies on peripheral blood CD8^+^ T cells indicate that aging is associated with a more senescent immune profile predictive of mortality [12], including a decrease in frequency of naïve CD8^+^ T cells and concurrent increase in oligoclonal expansions of activated memory/effector cells [5, 20–22], including increases in total frequency of CD28^-^ [23, 24] and CD57^+^ populations [25].

Our results indicate that in our healthy, relatively young cohort within each individual, on average, gut CD8^+^ T cells have a significantly more differentiated, activated profile than their peripheral blood counterparts. Nevertheless, certain phenotypic and functional features suggest that gut cells are not more senescent *per se*. Additionally, whereas blood cells show significantly increased activation and homeostatic proliferation with age, the only effect of age on gut cells is a further proportional shift from naïve to memory cells. Taken together, the data suggest that the presence of a large proportion of highly-differentiated, activated, proliferating memory CD8^+^ T cells in the gut may reflect a region-specific homeostasis that is preserved through young/middle aged adult life.

## Materials and methods

### Study subjects

This study was approved by the University of California, Los Angeles Medical Institutional Review Board and each participant provided written, informed consent per the approved protocol (UCLA IRB # 11–022238 and 11–001592). This report describes data gathered from blood and gastrointestinal (colorectal) mucosal biopsy (gut) samples collected from a total of 39 self-reported healthy participants. All participants are relatively ‘young’ (range: 24–67 years old; mean age: 40.8; male (n = 35), female (n = 3), MTF (n = 1)), and are a subset of the participants in a larger study aimed at examining the comparative effects of aging and treated vs. untreated HIV-infection (AG032422 PI: Effros).

### Collection of peripheral blood mononuclear cells (PBMC)

Human peripheral blood samples were acquired by standard venipuncture immediately prior to endoscopy; 70cc of peripheral blood to be used for Ki-67 staining, telomerase measurement and the proliferation assay were collected in seven 10ml Heparin tubes and immediately isolated by Ficoll gradient separation. Following Ficoll centrifugation, PBMC were washed with 1X PBS and resuspended in 10ml culture media (1X RPMI 1640, 15% FBS, 10mM HEPES, 2 mM glutamine, 50 IU/ml penicillin/streptomycin, 500 μg/ml Zosyn [piperacillin-tazobactam], 1.25ug/ml amphotericin B). Viable PBMC concentration was calculated via trypan blue exclusion.

An additional 8cc of whole blood were collected in two 4ml EDTA tubes: one was transferred to the flow cytometry research laboratory for T-cell immunophenotyping and the other was transferred to the UCLA CLIA certified Clinical Immunology Research laboratory for a White Blood Cell (WBC) with absolute lymphocyte count, using a SYSMEX XT 1800i hematology analyzer. Twenty-eight donors were tested for presence of CMV antibodies in WB at the UCLA CLIA certified Clinical Immunology Research laboratory.

### Collection of mucosal mononuclear cells (MMC)

Mucosal biopsy samples were collected as previously described [26]. Briefly, rectosigmoid biopsies were endoscopically acquired by flexible sigmoidoscopy between 10cm and 30cm from the anal verge. Biopsies were obtained by the use of large cup endoscopic biopsy forceps (Microvasive Radial Jaw #1589, Boston Scientific, Natick, MA). At each biopsy procedure, 30 specimens were collected into two 50ml tubes containing 20-25ml of RPMI medium with 7.5% fetal calf serum (FCS) (R-7.5), l-glutamine, amphotericin-B (1.25ug/ml) and piperacillin-tazobactam (50ug/ml). Samples were transported to the laboratory within 2 hours of collection. Upon receipt, the transport media was aspirated and biopsies incubated in 20–25ml RPMI/7.5% FCS containing 0.5 mg/ml collagenase type II-S (sterile filtered) (clostridiopeptidase A from *Clostridium histolyticum*, Cat. #C1764, Sigma-Aldrich, St. Louis, MO) for 30 min in a 37°C water bath, with intermittent shaking. Tissue fragments were further disrupted by forcing the suspension five to six times through a 30-cm^3^ disposable syringe attached to a blunt-ended 16-gauge needle (Stem Cell Technologies, Vancouver, BC). The entire suspension was then passed through a 70mm sterile plastic strainer (Falcon # 352350) to remove free cells and concentrate the remaining tissue fragments. Free cells were immediately washed twice in R-7.5 medium to remove excess collagenase, and tissue fragments were returned to a 50-ml conical tube. The entire procedure, including 30-min collagenase incubations, was repeated two additional times until tissue fragments were no longer intact (~ 2–3 hours duration). The isolated mucosal mononuclear cells (MMC) were combined and resuspended in 5ml of RPMI medium containing 10% FCS, amphoterin-B (1.25ug/ml) and Zosyn (50ug/ml). The MMC were used for flow cytometry and functional studies. Absolute CD3 T cell numbers were approximated using TRUcount™ beads. In a total of 135 donors to date, (including the samples in this study), the average recovery of MMC from 30 biopsies was 6.2x10^6^ CD3^+^ T cells, with a standard deviation of 3.5x10^6^ and range of 1.6x10^6^-25.0x10^6^.

### Cell surface phenotying

Cell surface staining was performed on 100ul aliquots of EDTA treated whole blood (WB) and freshly isolated MMC. WB samples were stained for 30’ at room temperature followed by treatment with ammonium chloride lysing solution to remove erythrocytes. MMC were stained for 30’ at 4° and washed twice with 1 ml PBS staining buffer (1X phosphate buffered saline with 2% heat inactivated newborn calf serum and 0.1% sodium azide), each wash was followed by centrifugation at 300xg for 5’ followed by careful aspiration of the supernatant. Samples were re-suspended in 500ul PBS staining buffer for acquisition by flow cytometry.

The staining panel consisted of 7 tubes with CD3 PerCP, CD4 (PE-CY7 or APC-H7)/ CD8 APC-H7. T-cells were investigated for expression of CD57, HLA-DR or CD45RO in the FITC parameter, CD28, CD38, CD31, CD8β, or CD25 in the PE parameter, CD45RA PE-CY7, and PD-1, CD27, CD28, CD56 in the APC parameter. All monoclonal antibodies (mAbs) were purchased from BD Biosciences (San Jose, CA) except: CD45RO was purchased from DAKO (Carpenteria, CA), PD-1 and CD27 were purchased from eBioscience (San Diego, CA). Appropriate gamma controls were run as a guide for cursor placement.

### Intracellular Ki-67 staining

To measure intracellular Ki-67, 0.5x10^6^ CD3^+^ MMC and 1.0x10^6^ PBMC, as determined by BD TruCount™, were stained per tube. MMC and PBMC were stained with the following antibodies: Ki-67 PE Antibody Set (*BD Pharmingen™*) containing mouse anti-human Ki-67 antibody and matching IgG1 isotype control, CD45RA FITC, CD3 Per-CP, CD4 PE-CY7, CD28 APC and CD8 APC-H7. Intracellular staining of PBMC and MMC was performed using the e-Bioscience Foxp3 Staining Buffer Set. After cell surface staining, samples were re-suspended in 1 mL of Fixation/Permeabilization working solution and incubated for 30’ at 4° followed by one wash in 1 ml of PBS staining buffer. Cells were then subjected to 2 washes in 2mL of 1X Permeabilization Buffer and centrifuged at 300xg for 7’ followed by careful aspiration of the supernatant. Cells were incubated for 10’ in 2% normal mouse serum after which the IgG1 isotype control was added to Tube 1 and Ki-67 added to Tube 2. Samples were incubated for an additional 30’ at 4° and washed 2 times with permeabilization buffer before being re-suspended in 500ul PBS staining buffer for acquisition by flow cytometry.

### Flow cytometry analysis

Samples were acquired on a 2 laser BD FACSCanto™ flow cytometer using FACSDiva™ software. Cytometer performance was optimized each day using BD™ Cytometer Setup and Tracking (CS&T) Beads. In addition, gluteraldehyde fixed chicken red blood cells (Biosure, Grass Valley, CA) were targeted daily to standardize for fluorescence. Single stained WB tubes were run to establish compensation settings using the manufacturer’s automated program for compensation set-up, and optimized as necessary. The cytometer stop counter was set to count 50,000 CD3^+^ events. Although the number of MMC counted did not always reach that threshold, the average across all donors was 40,000 for cell surface staining; Ki-67 staining averaged >18,000 for both CD4^+^ and CD8^+^ events. Further subsetting of Ki-67^+^, CD4^+^ or CD8^+^ was performed on a minimum of 100 Ki-67^+^ events.

For sample analysis, a primary gate was set on CD3 positive events using a CD3 vs. side scatter (SSC) dot plot. CD3 positive events were then passed through a forward scatter (FSC) vs. SSC plot to omit low FSC events representing dead and dying cells that stained non-specifically for all markers, this step was critical for MMC but not relevant for WB. CD3 positive events were displayed as either CD3 vs. CD4 or CD8 or alternatively, CD8 vs. CD4 to select CD4/CD8 subsets for further interrogation.

### Fluorescence-activated cell sorting (FACS) of blood and gut CD3^+^ cells

Purified T-cells (CD3^+^CD19^-^) were sorted from PBMC and MMC preparations stained with CD45 PerCP, CD3 FITC, CD19 PE, CD4 APC and CD8 APC-H7 (BD Biosciences, San Jose, CA.). Sorting was performed on a BD FACSAria™ flow cytometer. Instrument performance was validated with CS&T and BD™Accudrop beads. Lymphocyte sort gates were set using a CD45 vs. SSC gate and a FCS vs. SSC gate. Total T-cells were selected as CD3^+^ and CD19^-^. Aliquots of 320,000 cells per vial were sorted for the telomerase assay. Post sort analysis indicated T-cell purities of 97–100%. To reduce the degree of cellular clumping during the sort, MMC samples were re-suspended in PBS staining buffer at a dilute concentration of 1–2 million cells per ml and passed through 35um strainer capped tubes (BD, Franklin Lakes, NJ). PBMC were processed similarly except the concentration was 5-10x10^6^/ml. Presort and sort collection vials were kept chilled (4°C). The sorted cells were washed in 1X PBS and pelleted into 1 ml Eppendorf tubes as dry cell pellets for storage at -80°C until batch processed for telomerase activity measurement.

### Telomerase activity

Telomerase activity was determined using a modified version of the Telomere-repeat amplification protocol (TRAP), as previously described [27]. Briefly, for each donor, sorted cell pellets of 0.32×10^6^ purified CD3^+^ cells were lysed in 40 μl of M-PER Protein Extraction Reagent lysis buffer (Pierce, Rockford, IL). To control for intersample cell number variance, activity was normalized by nucleic acid concentration, which was determined using a Nanodrop 1000 (ThermoScientific, Wilmington, DE). The endogenous telomerase present in the cell extract adds telomeric repeats to the telomerase substrate (TS), a nontelomeric oligonucleotide. The extension products are then amplified several-fold by the polymerase chain reaction (PCR) carried out by *Taq polymerase* using a Cy-5-labeled forward primer (known as TS: 5′-/5Cy5/AATCCGTCGACGCAGA GTT) as a substrate for telomerase-mediated addition of TTAGGG repeats, and an anchored reverse primer (ACX*5′-GCGCCGCTTACCCTTACCCTTACCCTAACC-3′*). Following amplification, each sample was mixed with 25 μl of Bromothenol Blue loading dye and 25 μl of sample and loading dye were loaded and run using 10% nondenaturing PAGE in 1X TBE buffer. Gels were run at approximately 300 V for 80 min. Two replicates of each sample were evaluated, with the average fluorescence value used. All values were normalized to a standardized cell number of a telomerase-positive control cell line (Jurkat). Gels were scanned on a STORM 865 (GE Healthcare, Piscataway, New Jersey, USA) and quantified using the software ImageQuant 5.2, which integrates signal intensity over the telomere length distribution on the gel as a function of molecular weight (GE Healthcare).

### Proliferation assay

Proliferation status of CD8^+^ T cells was determined using a recently developed double-label CFSE/BrdU system on 5-day cultures of blood and gut derived mononuclear cells stimulated with CD3/CD28/CD2 antibodies, as previously described in extensive detail [28]. On day 5, live CD8^+^ T cells from blood and gut cultures that had undergone proliferation following stimulation were identified as CFSE^low^BrdU^+^. Live cells that had not undergone any proliferative divisions were CFSE^hi^BrdU^-^. Day 5 mean $\frac{proliferations}{cell}$ was determined for each donor using mean CFSE fluorescence value of non-dividing (CFSE^hi^BrdU^-^) and dividing (CFSE^low^BrdU^+^) populations; formula: $\frac{proliferations}{cell}=\log\left(\frac{mean\;CFSE\;non-dividing\;population}{mean\;CFSE\;dividing\;population}\right)÷log(2)$. The percentage of cells ≥ 4 divisions for each donor was determined by tabulating the percentage of total dividing (CFSE^low^BrdU^+^) cells whose CFSE value was ≥ 4 population halvings starting from the mean non-dividing (CFSE^hi^BrdU^-^) population CFSE.

### Statistical analysis

The analysis involved intra-individual comparisons of blood vs. gut lymphocytes. Before analysis began, data were examined for outliers and normality using histograms and qq plots. Because of the relatively small sample size and possible non-normality, we used non-parametric tests for comparisons. Paired observations of blood and gut from each individual were tested using the Wilcoxon signed rank test. To test for age-effects, we used generalized linear models using SAS V9.3 (Cary, NC). All tests were 2-sided and p-values < 0.05 were considered significant. ** p < 0.05, *** p < 0.005.

## Results

### Comparison of senescence-related markers in blood and gut

We initially confirmed that, on average, within each individual, there was no significant difference between blood and gut with respect to the percentage of CD8^+^ cells within the total CD3^+^ T cell population (mean of 32.7% versus 31.5% respectively, n = 39, p = 0.42, Table 1). (Note: in all comparisons blood values are listed first). Within the CD8^+^ T cell subset, we first evaluated expression of CD45RA and CD28. Use of these two markers is an accepted convention to distinguish T cell differentiation [29, 30], and differential expression of these markers (especially loss of CD28) on the surface of CD8^+^ T cells is associated with immune senescence, morbidity and mortality [2, 9, 12, 31–34]. Our data show that in the CD8^+^ T cell compartment, the gut had a significantly higher proportion of CD45RA^-^ (mean of 29.3 versus 88.5, n = 39, p<0.0001, Table 1) and CD28^-^ (mean of 30.1 versus 46.8, n = 39, p<0.0001, Table 1) cells. Looking at these markers together, there was a lower proportion of CD4RA^+^CD28^+^ naïve (mean of 47.9 versus 7.7, n = 39, p<0.0001, Table 1), and higher proportion of CD45RA^-^CD28^+^ early memory (mean of 24.5 versus 45.4, n = 39, p<0.0001, Table 1) and CD45RA^-^CD28^-^ late memory (mean of 4.8 versus 43.0, n = 39, p<0.0001, Table 1) CD8^+^ T cells. These results accord with earlier studies indicating that the gut CD8^+^ compartment is more differentiated than blood, presumably due to chronic antigenic stimulation [35]. Consistent with earlier studies [36, 37], the proportion of CD8α^+^β^-^ cells in the gut was greater than in the blood, with values approaching statistical significance (mean of 3.2 versus 4.2, n = 33, p = 0.06, Table 1).

**Table 1:** Comparison of senescence-related markers in blood and gut.

	Blood [mean (st. dev)]	Gut [mean (st. dev)]	p-value for the paired difference, n
**Percentage of CD8**^**+**^ **on CD3**^**+**^	32.7 (9.4)	31.5 (5.5)	0.4214, n = 39
**Percentage of CD8**^**+**^ **T cells**			
** CD45RA**^**-**^	29.3 (11.1)	88.5 (8.6)	<0.0001, n = 39***
** CD28**^**-**^	30.1 (15.1)	46.8 (12.8)	<0.0001, n = 39***
** CD45RA**^**+**^**CD28**^**+**^	47.9 (15.8)	7.7 (7.0)	<0.0001, n = 39***
** CD45RA**^**-**^**CD28**^**+**^	24.5 (10.3)	45.4 (11.6)	<0.0001, n = 39***
** CD45RA**^**-**^**CD28**^**-**^	4.8 (3.9)	43.0 (12.6)	<0.0001, n = 39***
** CD8α**^**+**^**β**^**-**^	3.2 (1.5)	4.2 (2.5)	0.06, n = 33
** PD-1**^**+**^	24.0 (12.2)	24.9 (14.1)	0.35, n = 34
** PD-1**^**+**^ **on CD45RO**^**+**^	40.9 (14.9)	30.7 (15.5)	0.0016, n = 34***
** CD57**^**+**^	31.0 (15.0)	11.2 (6.0)	<0.0001, n = 39***
** CD28**^**-**^**CD57**^**+**^	25.3 (14.1)	2.7 (1.4)	<0.0001, n = 39***
** CD25**^**+**^	5.9 (5.0)	0.9 (0.8)	<0.0001, n = 34***
** CD25**^**+**^ **on CD45RO**^**+**^	11.9 (8.7)	1.0 (0.9)	<0.0001, n = 34***

Intra-individual differences between blood and gut senescence-related markers were assessed using the Wilcoxon Signed Rank test for paired data. P-values < 0.05 were considered significant.

***, p<0.005.

Interestingly, the proportion of RO^+^ memory CD8^+^ T cells expressing PD-1 (the inhibitory receptor of programmed death), whose expression is associated with impaired T cell responses to viral infections in mice and humans and is also predictive of disease progression in HIV infected persons [38–40], was significantly *higher* in the blood versus the gut (mean of 40.9 versus 30.7, n = 33, p = 0.0016, Table 1). There was no significant difference in PD-1 expression on total CD8^+^ T cells (mean of 24.0 versus 24.9, n = 33, p = 0.35, Table 1). Additionally, the senescence-associated marker, CD57, had significantly *lower* expression in gut compared to blood (mean of 31.0 versus 11.2, n = 39, p<0.0001, Table 1). Within the total blood lymphocyte population, CD8^+^CD57^+^ cells have the shortest telomere length, have undergone more proliferative generations, and have a relatively high cytotoxic potential and poor proliferative capacity [25, 41]. Also, their frequency *in vivo* is correlated with conditions associated with immune dysregulation, such as CMV and HIV infection, and autoimmunity [42].

Surprisingly, the blood senescence-associated phenotype, CD8^+^CD28^-^CD57^+^ [43], was found to be nearly non-existent in the gut (mean of 25.3% versus 2.7%, n = 39, p<0.0001, Table 1). Additionally, CD25, which is constitutively expressed on some blood CD8^+^ T cells of elderly persons with a central memory-like phenotype and believed to be involved in protective humoral immunity [44, 45], had much lower expression among total CD8^+^ T cells in gut (mean of 5.8 versus 0.9, n = 33, p<0.0001, Table 1), especially in the CD45RO^+^ subset (mean of 11.9 versus 1.0, n = 33, p<0.0001, Table 1). Significantly lower expression of CD57, PD-1 on CD45RO^+^ memory cells, and CD25 on CD8^+^ T cells in gut indicates that although more highly differentiated, cells in this compartment may not be more functionally senescent.

### Comparison of markers of activation and homeostatic proliferation in blood and gut

Chronic activation and homeostatic proliferation contribute to increased cell turnover and differentiation, which can ultimately lead to replicative senescence. In blood, the MHC Class II surface receptor HLA-DR is upregulated in concert with T cell activation, and expression of the multifunctional enzyme CD38 on CD8^+^ T cells is associated with chronic activation and aging [46, 47]. Our data show that the gut CD8^+^ T cell population has a significantly higher proportion of cells that were HLA-DR^+^CD38^+^ as compared to the blood (mean of 3.8 versus 7.1, n = 39, p = 0.01, Table 2). Similarly, the gut also showed greater homeostatic proliferation, assessed by intracellular staining of Ki-67 (mean of 2.6 versus 4.3, n = 38, p = 0.02, Table 2).

**Table 2:** Comparison of markers of activation and homeostatic proliferation in blood and gut.

	Blood [mean (st. dev)]	Gut [mean (st. dev)]	P-value for the paired difference, n
**Percentage of CD8**^**+**^ **T cells**			
** HLA-DR**^**+**^**38**^**+**^	3.8 (2.5)	7.1 (4.6)	0.01, n = 39**
** Ki-67**^**+**^	2.6 (1.5)	4.3 (3.4)	0.02, n = 38**
** Ki-67**^**+**^**CD45RA**^**-**^**CD28**^**+**^	42.7 (14.8)	31.5 (18.8)	0.0009, n = 38***
** Ki-67**^**+**^**CD45RA**^**-**^**CD28**^**-**^	9.5 (6.2)	62.0 (18.8)	<0.0001, n = 38***
**CD3**^**+**^ **telomerase activity**	2.0 (2.9)	2.9 (1.4)	0.08, n = 20

Intra-individual differences between blood and gut activation and homeostatic proliferation-related markers were assessed using the Wilcoxon Signed Rank test for paired data. P-values < 0.05 were considered significant.

**, p<0.05

***, p<0.005.

Interestingly, when we gate on CD8^+^KI-67^+^, there is a higher percentage of CD45RA^-^CD28^+^ early memory proliferating cells in blood vs. gut (mean of 42.7 versus 31.5, n = 38, p = 0.0009, Table 2), but there is a greater than 6-fold increase in CD45RA^-^CD28^-^ late memory proliferating cells in gut versus blood (mean of 9.5 versus 62.0, n = 38, p<0.0001, Table 2). Due to limiting cell numbers, total CD3^+^ T cells, rather than the CD8^+^ subset, were evaluated for *ex vivo* telomerase activity. Telomerase is a holoenzyme critical for extended cellular proliferation[48] which has been shown to be upregulated in recently activated T cells [49, 50]. We found that gut had higher normalized relative telomerase activity compared to blood, although the value did not reach significance (mean of 2.0 versus 2.9, n = 20, p = 0.08, Table 2).

### Proliferative capacity of stimulated CD8 T cells in 5 day cultures

**Fig 1:**
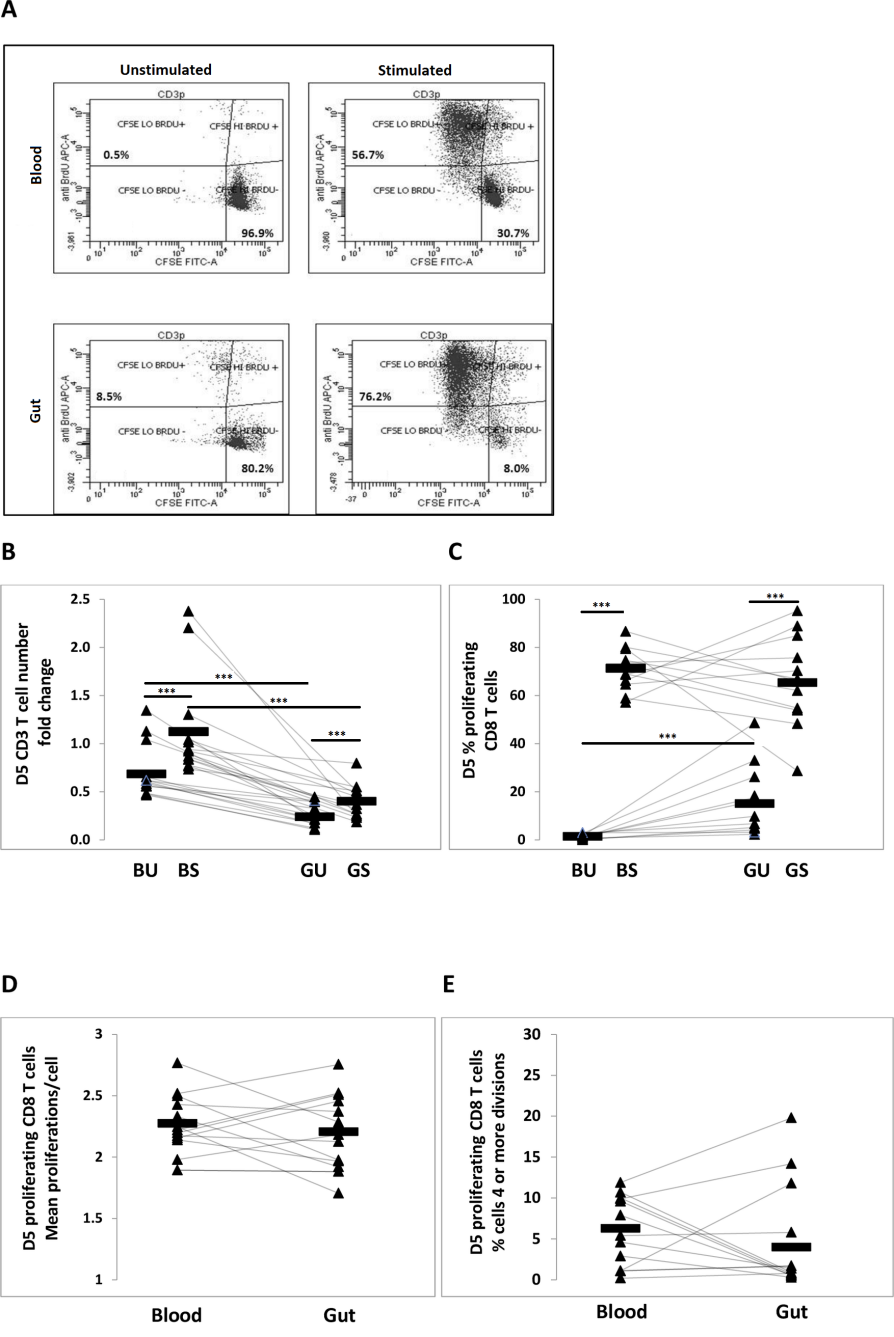
Proliferative capacity of stimulated CD8 T cells in 5 day culture. Proliferation of CD3^+^/CD8^+^ T cells was determined using a recently developed double—label CFSE/BRDU system on 5-day cultures of blood and gut derived mononuclear cells stimulated with CD3/CD28/CD2 antibodies. All cultures include 1.0x10^6^ blood or gut mononuclear cells and 0.5x10^6^ irradiated autologous PBMC feeders. **A.** Representative bivariate plots from blood- and gut-derived cultures (stimulated culture and unstimulated control). CFSE-FITC (x-axis) versus BrdU-APC (Y-axis) gating used to enumerate replicating (CFSEloBrdU^+^) verus non-replicating (CFSEhiBrdU^-^) CD8^+^ population. **B.** Day 5 CD3^+^ T cell number fold change (n = 13) **C.** Percentage of CD8^+^ T cells that are proliferating on Day 5 (n = 13). **D.** Mean $\frac{proliferations}{cell}$ of proliferating CD8^+^ T cells (n = 13). **E.** Percentage of highly proliferative (≥ 4 divisions) CD8^+^ T cells (n = 13). Intra-individual differences between blood and gut were assessed using the Wilcoxon Signed Rank test for paired data. P-values < 0.05 were considered significant. **, p<0.05, ***, p<0.005.

A defining feature of senescent T cells *in vitro* is inability to proliferate in response to stimulation [7], and, importantly, poor T cell proliferative ability *ex vivo* has been implicated as part of an immune risk profile (IRP) predictive of morbidity and mortality in the elderly [51]. We tested the proliferative capacity of CD3/CD28/CD2-stimulated blood and gut derived mononuclear cells in 5-day cultures. For each donor, both stimulated and unstimulated (control) cultures were evaluated, using our recently developed double-label CSFE/BrdU system [28]. Briefly, proliferating cells registered as CFSE^lo^BrdU^+^ and non-proliferating cells CFSE^hi^BrdU^-^ (Fig 1A).

For both blood and gut, stimulated cultures had significantly greater CD3^+^ T cell yields (calculated via Trucount™) on Day 5 compared to un-stimulated cultures (blood: mean fold-change of 1.2 (stim) versus 0.68 (unstim), n = 13, p<0.005; gut: mean fold-change of 0.4 (stim) versus 0.2 (unstim), n = 13, p<0.005, Fig 1B). Additionally, on Day 5, gut cultures contained significantly fewer total CD3^+^ T cells than blood in both unstimulated (mean fold change of 0.68 versus 0.23, n = 13, p<0.005, Fig 1B) and stimulated (mean fold change of 1.2 versus 0.4, n = 13, p<0.005, Fig 1B) cultures. As CD3^+^ T cell survival on Day 5 was approximately three times higher in blood compared to gut in both stimulated and control compartments, these results indicate that CD8^+^ T cells derived from the gut have a reduced ability to survive in culture, irrespective of simulation.

On Day 5, for both blood and gut, the percentage of proliferating (CFSE^lo^BrdU^+^) CD8^+^ T cells in the stimulated wells was significantly greater than in the unstimulated wells (blood: mean percentage of 71.2 (stim) versus 1.4 (unstim), n = 13, p<0.005; gut: mean percentage of 65.4 (stim) versus 15.1 (unstim), n = 13, p<0.005, Fig 1C). On day 5 there was no difference between blood and gut in the proportion of live CD8^+^ T cells proliferating in stimulated cultures (mean percentage of 71.2 versus 65.0, n = 13, p = 0.41, Fig 1C). In unstimulated culture wells, gut exhibited a significantly higher proliferation profile (mean percentage of 1.4 versus 15.1, n = 13, p<0.005, Fig 1C), consistent with Ki-67 and telomerase data indicating a higher proportion of gut T cells are proliferating *ex vivo*.

We observed that the average number of rounds of proliferation (determined via CFSE dilution) among proliferating CD8^+^ T cells in stimulated cultures on Day 5 did not differ significantly between blood and gut (mean of 2.3 divisions versus 2.2, n = 13, p = 0.49, Fig 1D). There was also no significant difference in percentage of cells that had undergone 4 or more divisions (mean of 5.8 versus 4.5, n = 13, p = 0.49, Fig 1E).

### Influence of age on CD8 T cells in blood vs. gut

**Fig 2:**
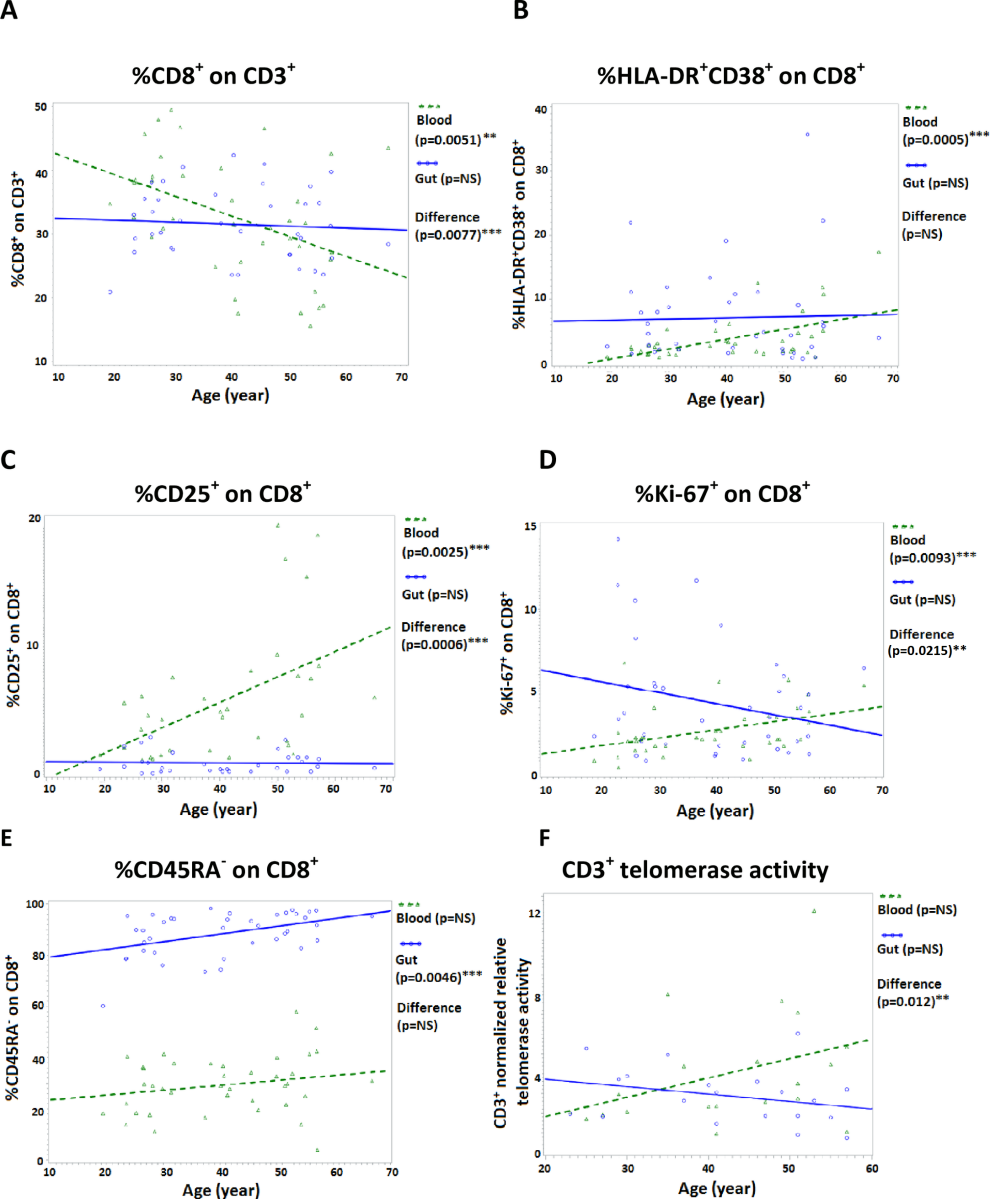
Significant age-effect differences in blood vs gut T lymphocyte populations. Age-effect in blood and gut T lymphocyte parameters, and intra-individual age-effect difference between compartments, was tested using generalized linear models using SAS v9.3. For (**A-E**), blood and gut T cell WB aliquots were phenotyped by multi-color flow cytometry and analyzed (see methods). Differential age-effect data for proportion of **(A)** CD8^+^ on CD3^+^ (n = 39), **(B)** HLA-DR^+^CD38^+^ on CD8^+^ (n = 39), **(C)** CD25^+^ on CD8^+^ (n = 34), **(D)** Ki-67^+^ on CD8^+^ (n = 39), and **(E)** CD45RA^-^ on CD8^+^ (n = 39) is shown. For **(F)**, relative telomerase activity of 0.32x10^6^ blood and gut purified CD3^+^ T cells from each donor was determined via the PCR-based TRAP protocol, normalized to a standardized cell number of a telomerase-positive control cell line (Jurkat), and differential age-effect was determined (n = 20). P-values < 0.05 were considered significant. **, p< 0.05, ***, p<0.005.

We sought to determine possible age-effect within the two compartments in this cohort of relatively young/middle age subjects. In peripheral blood, older age had a significant impact on the following: proportion of CD8^+^ on total CD3^+^ (decrease, p = 0.0051), CD8^+^HLA-DR^+^CD38^+^ (p = 0.0005), CD8^+^CD25^+^ (p = 0.0025), CD8^+^CD25^+^ on RO^+^ memory (p = 0.0011) and CD8^+^Ki-67^+^ (p = 0.0093) (Fig 2A–2D). The observed age-effect is consistent with peripheral blood CD8^+^ T cells becoming more activated and proliferating more during aging. Increased proportion of CD25^+^ cells with aging is consistent with previous studies indicating this activation marker is preferentially expressed in elderly persons [44, 45].

In gut mucosa cells, there was a significant age-effect increase in CD8^+^CD45RA^-^ memory cells (p = 0.0046) (Fig 2E), and a corresponding decrease in CD8^+^CD45RA^+^CD28^+^ naïve cells (p = 0.0088) (data not shown), indicating further differentiation in this compartment from naïve to memory populations during aging.

Directly comparing intra-individual blood and gut compartments with respect to age, several compartment-specific age effects were noted. Specifically, there was a significant age-effect difference between blood and gut in the following: proportion of CD8^+^ on total CD3^+^ (p = 0.008), CD8^+^CD25^+^ (p = 0.0006), CD8^+^CD25^+^ on RO^+^ memory (p = 0.0007), CD8^+^Ki-67^+^ (p = 0.02), and CD3 *ex-vivo* telomerase activity (p = 0.01) (Fig 2A, 2C, 2D and 2F). There was also a non-significant, but noticeable, difference in the following: proportion of CD8^+^PD-1^+^ on RO^+^ memory (p = 0.11) and CD8^+^HLA-DR^+^CD38^+^ (p = 0.16) (data not shown). In these cases, the majority of the difference is attributed towards a strong age-effect in peripheral blood, with little or no age-effect in the mucosal compartment. S1 Table shows differential age-effect in blood and gut and intra-individual age-effect difference between compartments for all parameters tested in the cohort.

## Discussion

To our knowledge, this is the first study comparing intra-individual differences in multiple parameters of human T cells in two distinct immune compartments, namely the peripheral blood and gut mucosa. The gut is a primary line of defense that routinely faces antigenic challenges, as opposed to the blood, which is primarily a conduit for trafficking lymphocytes. We therefore hypothesized that gut CD8^+^ T cells would have undergone a greater degree of antigenic driven proliferation and differentiation, and be more senescent and activated than their blood counterparts. This hypothesis is consistent with early studies on intestinal lamina propria T cells [15] and previous unpublished observations from our laboratory, indicating that gut CD8^+^ T cells are predominately CD45RA^-^ and more activated than their blood counterparts. This notion is also consistent with rodent data indicating age-associated alterations in the GALT similar to peripheral blood immune senescence in humans. These include changes in CD8^+^ T cell distribution [52], decrease in naïve CD4^+^ T cells and dendritic cells in Peyers patches [53], defects in mucosal IgA and IgG secretion [54, 55], and defects in T cell function and vaccine immune responses [56–58].

The key finding of our analysis is that, although CD8^+^ T cells within the gut have characteristics associated with greater activation and differentiation compared to blood, other features suggest that these changes are not indicative of cellular senescence *per se*. Additionally, at least in this relatively young/middle-age population, whereas CD8^+^ blood cells show elevated expression of markers for activation and proliferation with age, gut cells do not; instead, they demonstrate a significant further homeostatic shift from naïve to memory status.

Consistent with previous aforementioned studies, intra-individual comparisons of blood versus gut (rectosigmoid colon) CD8^+^ T cell parameters indicate gut cells are generally more differentiated and activated. Thus, the gut has a relatively smaller proportion of CD45RA^+^CD28^+^ naïve CD8^+^ cells, and higher proportion of CD45RA^-^CD28^+^ early memory, CD45RA^-^CD28^-^ late memory, HLA-DR^+^38^+^ activated, and Ki-67^+^ proliferating cells. Additionally, gut exhibited a non-significant increase in CD3^+^ T cell *ex vivo* telomerase activity (p = 0.08).

However, the study also highlights several unexpected compartment-specific differences in phenotypic markers previously associated with aging and cellular senescence in peripheral blood. Several groups have previously identified CD8^+^CD28^-^CD57^+^ cells as very late-differentiated, presumably due to chronic antigenic stimulation and homeostatic proliferation, with several features indicative of cellular senescence, including short telomere lengths, poor proliferative potential, and high cytotoxic potential [43, 59, 60]. Our data demonstrate that in peripheral blood, 30.1% of CD8^+^ cells are CD28^-^ and 25.3% of are CD28^-^CD57^+^, indicating a high concurrence of the two markers, as expected. However, in gut, whereas 46.8% of cells are CD28^-^, only 2.7% are CD28^-^CD57^+^. The total proportion of CD57^+^ on CD8 T cells is also significantly lower in the gut (11.2%) than blood (31.0%). Additionally, the expression of PD-1, a marker of cellular exhaustion linked to immune dysfunction caused by chronic viral infections [40], is significantly lower on RO^+^ memory CD8^+^ cells in gut (30.7%) compared to blood (40.9%). Finally, constitutive CD25 expression on CD8 T cells, which is implicated in protective humoral immunity in the elderly [45], is expressed on 11.9% of blood RO^+^ CD8^+^ T cells, but only 0.9% of gut cells.

The above data indicate that although mucosal gut CD8^+^ T cells are predominantly memory and are more differentiated and activated *ex vivo*, there may be mechanisms preventing the acquisition of other features associated with cellular senescence/dysfunction. Consistent with this notion, we observed that in gut 62.0% of Ki-67^+^ proliferating CD8^+^ cells were CD45RA^-^CD28^-^, a phenotype correlated with *reduced* proliferative capacity in blood. It has been previously documented that blood CD8^+^CD28^-^ T cells represent a heterogeneous population *in vivo*, including cells that still have modest replicative potential [8, 43]. However, such a high proportion of cycling CD8^+^ cells in gut mucosa being CD45RA^-^CD28^-^ indicates these observations might underestimate proliferative potential of late-differentiated CD8^+^ cells in this, and other, tissues. Further supporting this notion, on Day 5 following in *vitro* stimulation, there was no qualitative difference in proliferative dynamics of gut vs. blood CD8^+^ T cells (Fig 1C–1E), despite the presence of a significantly greater proportion of CD8^+^CD28^-^ T cells in the gut.

One possible reason that gut T cells may not develop functional senescence, despite significantly higher levels of presumably antigen-induced differentiation and activation relative to blood, is tolerization by the gut microenvironment. The intestinal immune system, unlike peripheral blood, is subject to constant exposure to not only pathogenic antigens, but also commensal bacteria and dietary antigens, and presumably has developed specialized features to induce adaptive tolerance [61–63]. In such a milieu, there may exist mechanisms to restrict local activated, antigen experienced CD8^+^ T cells from developing a senescent phenotype; as many senescent functional features, including high cytotoxic potential [8, 64] and proinflammatory cytokine secretion [65, 66], could contribute to gut mucosal inflammatory disorders such as Coeliac Disease and Crohn’s Disease. Future phenotypic and functional studies on gut CD8^+^ T cells—for example testing expression of *ex-vivo* INFγ, TNFα and perforin to further assess functional senescence [64], or measuring CD95 expression to assess tolerance induction through apoptosis [67, 68]—may help elucidate the underlying mechanism by which these cells may be protected from becoming functionally senescent/exhausted. In addition, future studies of gut CD8^+^ T cell differentiation and senescence in the context of chronic HIV infection could be informative in determining whether HIV induced severe disruption of gut homeostasis [69, 70], including severe depletion of resident Th17 CD4^+^ cell populations [71, 72] and chronic activation of resident CD8^+^ T cells [5, 73], could disrupt gut homeostasis and cause an increase in senescent/dysfunctional features on resident CD8^+^ T cells.

In peripheral blood of our relatively young/middle age cohort, increased age was associated with a significant increase in HLA-DR^+^38^+^ activated and Ki-67^+^ proliferating CD8^+^ T cells, and an increase in total CD3^+^ *ex-vivo* telomerase activity that approached statistical significance. These results are consistent with previous studies indicating that CD8^+^ T cells become more activated with age [5, 65, 74]. Interestingly, in peripheral blood of our study subjects, the proportion of CD8^+^ T cells (within the total T cell population) showed a significant decrease with age (Fig 2A). This observation contrasts with the OCTO/NONA studies on 80 and 90 year olds, where a *reduced* CD4:CD8 ratio (due to oligoclonal expansions of “senescent” CD8 T cells that were often specific for CMV) [9, 10, 75], was a component of the so-called immune risk phenotype (IRP) predictive of mortality risk [12]. Our data, showing a significant *increase* in CD4:CD8 ratio with age, is consistent with other studies [21, 76], one of which determined this increase to be due to disproportionate reduction in naïve CD8 T cells [21]. Our analysis of age-associated phenotypic and functional features was somewhat limited due to our intentional focus on subjects who were still relatively young, in order to eliminate potential confounding effects of chronic disease. Indeed, there was only one subject over age 60 in our cohort. This may explain our observation that in peripheral blood there was a significant decrease in CD8^+^ proportion and no significant age-effect in CD45RA^-^, CD28^-^ or CD57^+^ expression, whereas several earlier studies, which included persons >85 years, showed that these senescence-related markers increase significantly with age in the CD8^+^ T cell subset [20, 23, 24, 60]. It is possible that the age-effect of differential CD45RA/CD28/CD57 cell surface expression in peripheral blood is only noticeable in very elderly persons. This hypothesis is supported by data from Strindhall et al. [77] who in the follow-up HEXA study to the OCTO/NONA studies with a cohort all 66 years old, determined that 85% of participants had a positive CD4:CD8 ratio, and had a relatively high proportion of naïve and low proportion of very late differentiated CD8 cells.

In gut mucosal cells, the only significant effect of older age was an increase in proportion of CD45RA^-^ memory CD8^+^ T cells, and a concurrent decrease in proportion of the CD45RA^+^CD28^+^ naïve cells. The observation of no additional changes with age further supports the notion that gut cells do not follow the same age-related senescence dynamics as peripheral blood cells. Indeed, directly comparing intra-individual age-effect in blood vs. gut, there was a compartment-specific significant age-related difference within the following parameters: proportion of CD8^+^, CD25^+^ on CD8^+^, CD25^+^ on CD8^+^CD45RO^+^, Ki-67^+^ on CD8^+^, and CD3^+^ telomerase activity. In these parameters, the difference between compartments was due mainly to a strong age-associated increase in peripheral blood, and minimal age effects in gut.

As discussed above, an interesting finding of the longitudinal OCTO/NONA studies was that CMV seropositivity was a component of the IRP that predicted mortality in subjects > 80 years of age [12]; and that in elderly CMV^+^ individuals oligoclonal expansion of “senescent” late-differentiated CD8^+^ T cell populations were often specific for CMV antigens [9, 10, 75]. Subsequent studies indicate that latent CMV infection is significantly associated with a *general* increase in age-related alterations in T cell homeostasis in the CD4^+^ and CD8^+^ T cell compartments towards more late-differentiated phenotypes [20, 21, 29, 30, 78, 79], with many inter-study discrepancies. In our cohort we tested 28 subjects for presence of CMV antibodies at the time of sample collection, and 89% (n = 25) were found to be CMV^+^, consistent with known incidence rates in the general adult population [80]. Our known CMV^-^ population (n = 3) was too small to compare differential effects of CMV serostatus. However, we looked at intra-individual compartment and age-effect differences within the CMV^+^ cohort (S2 Table). Compared to the entire cohort, compartment and age-effect for the CMV^+^ cohort was similar for all variables with one exception; CD28^-^CD57^+^ expression on CD8^+^ T cells showed a significant age-related increase in gut in the CMV^+^ population (p = 0.01) not seen in the entire cohort. These results indicate our cohort is representative of the general population, where the majority of adult subjects have latent CMV infection. To determine whether CMV seropositivity has a significant impact on compartment and/or age-related T cell homeostasis, it would be necessary to recruit a much larger number of CMV^-^ subjects, and most-likely older subjects as well. However, a significant age-effect in gut (but not blood) in a very late-differentiated memory phenotype (CD8^+^CD28^-^CD57^+^ T cells) indicates CMV seropositivity may influence age-related compartment specific T cell homeostasis differentially.

Our initial analysis focused on establishing baselines for senescence-related parameters comparing blood and gut CD8^+^ T cell homeostasis in healthy young/middle-age subjects. This will, in turn, facilitate future research into how old age and chronic diseases perturb homeostasis differentially in the gut. Studying gut senescence in the context of HIV disease should be especially relevant, as it is believed that local immune activation, including that of HIV specific CD8^+^ T cells, and loss of infected CD4+ cells (especially Th17) early in the acute phase of the disease permanently compromises gut immune homeostasis [70, 72]. The next stage of our research will specifically address the effects of aging and HIV on the senescence profile in the gut. In addition, based on certain published age-related CD4^+^ T cell changes in blood [81–84], and our own preliminary observations on their early senescence within the gut, our future research will focus on both T cell subsets.

In conclusion, the current study establishes that gut CD8 T cells from the rectosigmoid colon of relatively young, healthy donors have many characteristics indicating that they are more differentiated and activated than their peripheral blood counterparts. However, certain other features (e.g., relatively low expression of CD57 and PD-1 on RO^+^ memory cells, a high percentage of cells undergoing homeostatic proliferation being effector memory, and minimal age-effect on all parameters) suggest that the gut is not more senescent *per se*. Further functional experiments on gut cells are required in order to clarify their functional status vis-à-vis cellular senescence. Importantly, this conclusion is based on only one gut region, which may not necessarily be representative of other regions of the GALT. In fact, pilot studies performed by our group indicate the small intestine has a lower CD4:CD8 ratio and a more ‘activated’ CD8 T cell phenotype than the colon, suggesting that our data may, in fact, be an underestimate of the overall difference between the mucosal and blood immune compartment.

## Supporting information

S1 Dataset Minimal dataset. Minimal dataset including all relevant data underlying the study’s findings, with removal of potentially identifying participant information.
(XLSX)

S1 Table Age-effect on blood and gut. Age-effect in blood and gut T lymphocyte parameters, and intra-individual age-effect difference between compartments, was tested using generalized linear models using SAS V9.3. P-values < 0.05 were considered significant. **, p< 0.05, ***, p<0.005.
(DOCX)

S2 Table Compartment and age-effect for CMV^+^ donors: Blood and gut. Intra-individual compartment and age-effect differences for CMV^+^ donors (n = 25, mean age 38.1 yrs.) was assessed (for CD8α^+^β^-^, n = 24, mean age 38.1 yrs.). Intra-individual compartment differences were assessed using the Wilcoxon Signed Rank test for paired data. Age-effect on blood and gut T lymphocyte parameters was tested using generalized linear models using SAS V9.3. P-values < 0.05 were considered significant. **, p< 0.05, ***, p<0.005.
(DOCX)
