# Supplementary material for: Distinct aging profiles of CD8+ T cells in blood versus gastrointestinal mucosal compartments
Source: PLoS One. 2017 Aug 23;12(8):e0182498. doi: 10.1371/journal.pone.0182498 (PMC5568404; doi:10.1371/journal.pone.0182498)
Supplement: S1 Table — Age-effect in blood and gut T lymphocyte parameters, and intra-individual age-effect difference between compartments, was tested using generalized linear models using SAS V9.3. P-values < 0.05 were considered significant. **, p< 0.05, ***, p<0.005. (DOCX) [file pone.0182498.s002.docx]

**S1 Table: Age-effect on blood and gut**

|  |  | **Age [mean (st. dev), n]** | **Age effect estimate** | **SE** | **p value** | **R2** |
| --- | --- | --- | --- | --- | --- | --- |
| Percentage of CD8^+^ on CD3^+^ | Blood | 40.6 (12.7), 39 | -0.3238 | 0.1088 | 0.0051^**^ | 0.1931 |
|  | Gut |  | -0.0312 | 0.0714 | 0.6646 | 0.0051 |
|  | Difference |  | -0.3247 | 0.1152 | 0.0077^**^ | 0.1546 |
| Percentage of CD8^+^ cells |  |  |  |  |  |  |
| CD45RA^-^ | Blood | 40.6 (12.7), 39 | 0.191 | 0.1402 | 0.1814 | 0.0478 |
|  | Gut |  | 0.2999 | 0.0994 | 0.0046^***^ | 0.1975 |
|  | Difference |  | -0.0888 | 0.1306 | 0.5004 | 0.0124 |
| CD28^-^ | Blood | 40.6 (12.7), 39 | 0.0724 | 0.1955 | 0.7063 | 0.0039 |
|  | Gut |  | -0.0507 | 0.1653 | 0.7607 | 0.0025 |
|  | Difference |  | 0.0897 | 0.2426 | 0.7138 | 0.0037 |
| CD45RA^+^CD28^+^ | Blood | 40.6 (12.7), 39 | -0.1376 | 0.2292 | 0.5525 | 0.0115 |
|  | Gut |  | -0.2592 | 0.0927 | 0.0088^**^ | 0.2012 |
|  | Difference |  | 0.1216 | 0.2307 | 0.6009 | 0.0089 |
| CD45RA^-^CD28^+^ | Blood | 40.6 (12.7), 39 | 0.20279 | 0.1286 | 0.1234 | 0.0629 |
|  | Gut |  | 0.2698 | 0.1439 | 0.0687 | 0.0868 |
|  | Difference |  | -0.0163 | 0.1632 | 0.9206 | 0.0003 |
| CD45RA^‑^CD28^-^ | Blood | 40.6 (12.7), 39 | -0.0199 | 0.05 | 0.8122 | 0.0015 |
|  | Gut |  | 0.0298 | 0.1637 | 0.8564 | 0.0009 |
|  | Difference |  | -0.0722 | 0.1658 | 0.6658 | 0.0051 |
| CD57^+^ | Blood | 40.6 (12.7), 39 | 0.174 | 0.1917 | 0.3698 | 0.0218 |
|  | Gut |  | 0.0469 | 0.0776 | 0.5489 | 0.0098 |
|  | Difference |  | 0.1473 | 0.1848 | 0.4308 | 0.0169 |
| CD57^+^CD28^-^ | Blood | 40.6 (12.7), 39 | 0.0943 | 0.1813 | 0.6059 | 0.0073 |
|  | Gut |  | 0.0184 | 0.0185 | 0.3249 | 0.0262 |
|  | Difference |  | 0.0815 | 0.1783 | 0.6502 | 0.0056 |
| CD8α^+^β^-^ | Blood | 40.7 (12.7), 33 | -0.0231 | 0.0283 | 0.2757 | 0.0382 |
|  | Gut |  | 0.0314 | 0.0352 | 0.3793 | 0.025 |
|  | Difference |  | -0.0636 | 0.0345 | 0.0749 | 0.0958 |
| CD25^+^ | Blood | 40.6 (12.7) 34 | 0.1989 | 0.0607 | 0.0025^***^ | 0.2514 |
|  | Gut |  | -0.0027 | 0.0113 | 0.8131 | 0.0018 |
|  | Difference |  | 0.2185 | 0.0568 | 0.0006^***^ | 0.3228 |
| CD25^+^ on CD45RO^+^ | Blood | 40.6 (12.7), 34 | 0.1608 | 0.0465 | 0.0016^***^ | 0.2714 |
|  | Gut |  | -0.0022 | 0.0086 | 0.798 | 0.0021 |
|  | Difference |  | 0.1659 | 0.0442 | 0.0007^***^ | 0.305 |
| PD-1^+^ on CD45RO^+^ | Blood | 40.6 (12.7), 34 | 0.1153 | 0.2098 | 0.5866 | 0.0096 |
|  | Gut |  | -0.0186 | 0.2196 | 0.9329 | 0.0002 |
|  | Difference |  | 0.1339 | 0.2256 | 0.5563 | 0.0113 |
| DR^+^38^+^ | Blood | 40.6 (17.7), 39 | 0.154 | 0.0401 | 0.0005^***^ | 0.2851 |
|  | Gut |  | 0.01884 | 0.0943 | 0.8427 | 0.0011 |
|  | Difference |  | 0.1486 | 0.1042 | 0.1621 | 0.0521 |
| Ki-67^+^ | Blood | 39.8 (12.9), 38 | 0.0477 | 0.01736 | 0.0093^**^ | 0.1734 |
|  | Gut |  | -0.0649 | 0.0423 | 0.1341 | 0.0612 |
|  | Difference |  | 0.1124 | 0.0468 | 0.0215^**^ | 0.1383 |
| CD45RA^-^CD28^+^ on Ki67^+^ | Blood | 39.8 (12.9), 38 | 0.3154 | 0.1833 | 0.0928 | 0.076 |
|  | Gut |  | 0.11329 | 0.2415 | 0.5854 | 0.0083 |
|  | Difference |  | 0.1824 | 0.2526 | 0.475 | 0.0143 |
| CD45RA^-^CD28^-^ on Ki67^+^ | Blood | 39.8 (12.9), 38 | -0.0844 | 0.0787 | 0.2908 | 0.0309 |
|  | Gut |  | -0.1275 | 0.2413 | 0.6005 | 0.0077 |
|  | Difference |  | 0.0431 | 0.2305 | 0.8695 | 0.0008 |
| CD3^+^ telomerase activity | Blood | 42.3 (11.1), 20 | 0.098 | 0.0563 | 0.0989 | 0.144 |
|  | Gut |  | -0.0371 | 0.0287 | 0.2159 | 0.0837 |
|  | Difference |  | 0.1315 | 0.0484 | 0.012^**^ | 0.3024 |

**S1 Table: Age-effect on blood and gut.** Age-effect in blood and gut T lymphocyte parameters, and intra-individual age-effect difference between compartments, was tested using generalized linear models using SAS V9.3. P-values < 0.05 were considered significant. ^**^, p< 0.05, ^***^, p<0.005.
