# Supplementary material for: Distinct aging profiles of CD8+ T cells in blood versus gastrointestinal mucosal compartments
Source: PLoS One. 2017 Aug 23;12(8):e0182498. doi: 10.1371/journal.pone.0182498 (PMC5568404; doi:10.1371/journal.pone.0182498)
Supplement: S2 Table — Intra-individual compartment and age-effect differences for CMV+ donors (n = 25, mean age 38.1 yrs.) was assessed (for CD8α+β-, n = 24, mean age 38.1 yrs.). Intra-individual compartment differences were assessed using the Wilcoxon Signed Rank test for paired data. Age-effect on blood and gut T lymphocyte parameters was tested using generalized linear models using SAS V9.3. P-values < 0.05 were considered significant. **, p< 0.05, ***, p<0.005. (DOCX) [file pone.0182498.s003.docx]

**S2 Table: Compartment and age-effect for CMV^+^ donors: blood and gut**

|  | **Compartment Effect** | | |  |
| --- | --- | --- | --- | --- |
|  | **Mean Blood** | **Mean Gut** | **p value (paired)** |  |
| Percentage of CD8^+^ on CD3^+^ | 33.4 | 30.4 | 0.1467 |  |
| Percentage of CD8^+^ T cells |  |  |  |  |
| CD45RA^-^ | 27.4 | 87.0 | <0.0001^***^ |  |
| CD28^-^ | 29.8 | 47.4 | <0.0001^***^ |  |
| CD45RA^+^CD28^+^ | 48.2 | 8.7 | <0.0001^***^ |  |
| CD45RA^-^CD28^+^ | 22.8 | 44.0 | <0.0001^***^ |  |
| CD45RA^-^CD28^-^ | 4.6 | 43.1 | <0.0001^***^ |  |
| CD8α^+^β^-^ | 3.3 | 4.4 | 0.0770 |  |
| PD-1^+^ | 20.9 | 25.2 | 0.0158^**^ |  |
| PD-1^+^ on CD45RO^+^ | 38.5 | 30.7 | 0.0080^**^ |  |
| CD57^+^ | 30.2 | 12.0 | <0.0001^***^ |  |
| CD28^-^CD57^+^ | 25.0 | 2.9 | <0.0001^***^ |  |
| CD25^+^ | 5.0 | 0.8 | <0.0001^***^ |  |
| CD25^+^ on CD45RO^+^ | 10.1 | 1.0 | <0.0001^***^ |  |
| DR^+^38^+^ | 3.6 | 6.7 | 0.0266^***^ |  |
| Ki-67^+^ | 2.6 | 5.1 | 0.0075^**^ |  |
| Ki-67^+^CD45RA^-^CD28^+^ | 41.0 | 32.0 | 0.0272^**^ |  |
| Ki-67^+^CD45RA^-^CD28^-^ | 10.0 | 61.6 | <0.0001^***^ |  |
| CD3^+^ telomerase activity | 3.5 | 3.3 | 0.63 |  |
|  | **Age Effect** | | | |
|  | **Blood correlation with age** | **p value** | **Gut correlation with age** | **p value** |
| Percentage of CD8^+^ on CD3^+^ | -0.4 | 0.045^**^ | -0.16 | NS |
| Percentage of CD8^+^ T cells |  |  |  |  |
| CD45RA^-^ | 0.23 | NS | 0.52 | 0.0081^**^ |
| CD28^-^ | 0.08 | NS | 0.05 | NS |
| CD45RA^+^CD28^+^ | -0.18 | NS | -0.43 | 0.0304^**^ |
| CD45RA^-^CD28^+^ | 0.25 | NS | 0.24 | NS |
| CD45RA^-^CD28^-^ | 0.06 | NS | 0.14 | NS |
| CD8α^+^β^-^ | -0.19 | NS | 0.29 | NS |
| PD-1^+^ | 0.19 | NS | 0.01 | NS |
| PD-1^+^ on CD45RO^+^ | -0.01 | NS | -0.11 | NS |
| CD57^+^ | 0.18 | NS | 0.17 | NS |
| CD28^-^CD57^+^ | 0.12 | NS | 0.50 | 0.0101^**^ |
| CD25^+^ | 0.57 | 0.0032^***^ | -0.08 | NS |
| CD25^+^ on CD45RO^+^ | 0.54 | 0.0052^**^ | -0.06 | NS |
| DR^+^38^+^ | 0.62 | 0.0008^***^ | -0.24 | NS |
| Ki-67^+^ | 0.37 | 0.0686 | -0.20 | NS |
| Ki-67^+^CD45RA^-^CD28^+^ | 0.09 | NS | 0.12 | NS |
| Ki-67^+^CD45RA^-^CD28^-^ | -0.14 | NS | -0.09 | NS |
| CD3^+^ telomerase activity | 0.32 | NS | -0.15 | NS |

**S2 Table: Compartment and age-effect for CMV^+^ donors: blood and gut.**  Intra-individual compartment and age-effect differences for CMV^+^ donors (n=25, mean age 38.1 yrs.) was assessed (for CD8α^+^β^-^, n=24, mean age 38.1 yrs.). Intra-individual compartment differences were assessed using the Wilcoxon Signed Rank test for paired data. Age-effect on blood and gut T lymphocyte parameters was tested using generalized linear models using SAS V9.3. P-values < 0.05 were considered significant. ^**^, p< 0.05, ^***^, p<0.005.
